# Tuberculous pleurisy mimicking *Mycoplasma pneumoniae* infection in a previously healthy young adult: A case report

**DOI:** 10.1097/MD.0000000000010811

**Published:** 2018-05-18

**Authors:** Daizo Yaguchi, Motoshi Ichikawa, Masato Shizu, Noriko Inoue, Daisuke Kobayashi, Naoyuki Imai, Masao Ito

**Affiliations:** a Department of Respiratory Medicine; b Department of Thoracic Surgery, Gifu Prefectural Tajimi Hospital, Tajimi, Gifu, Japan.

**Keywords:** adenosine deaminase, anti-mycoplasma antibody test, lymphocytic pleural effusion, *Mycoplasma pneumoniae* infection, tuberculous pleurisy

## Abstract

**Ratonale:**

Sometimes, pleural effusion accompanying an acute *Mycoplasma pneumoniae* infection or tuberculous pleurisy has similar analysis results. We report a case of tuberculous pleurisy which was initially diagnosed as acute *M pneumoniae* infection, which is of special interest because anti-Mycoplasma antibody results were positive, which served as a red herring.

**Patient concerns:**

A 20-year-old woman visited the outpatient emergency romm of our hospital for chief complaints of high fever, dry cough, and pleuralgia persiting for 2 days. Since anti-mycoplasma antibody test results were positive, we treated acute *M pneumoniae* infection and drained her pleural effusion. The condition tended to improve, but on day 16 postadmission, the acid-fast bacterial culture of the pleural effusion was positive for *Mycobacterium tuberculosis*.

**Diagnoses:**

Tuberculous pleurisy.

**Interventions:**

After the diagnosis, the patient received antituberculous drugs.

**Outcomes:**

She completed treatment with no noticeable adverse events, and the right pleural effusion disappered and diffuse right pleural thickening improved.

**Lessons:**

Exudative pleural effusion with lymphocyte dominance and a high adenosine deaminase level in *M pneumoniae* infection have been reported. Even though the condition suggests acute *M pneumoniae* infection, clinicians should be aware that tuberculous pleurisy and *M pneumoniae* infection can share similar clinical features, and should understand the usefulness and limitations of the anit-Mycoplasma antibody test.

## 1 Introduction

Tuberculous pleurisy constitutes 17% of tuberculosis cases in Japan.^[1]^ Patients with extrapulmonary tuberculosis have the highest incidence. Tuberculous pleurisy should always be considered in patients with pleural effusion. The onset patterns of tuberculous pleurisy and pulmonary tuberculosis differ. Diagnosis may be delayed, unless a clinician is aware that tuberculous pleurisy develops acutely and appears as a bacterial infection in one-third of patients.^[2]^ Furthermore, pleural effusion accompanying an acute *Mycoplasma pneumoniae* infection or tuberculous pleurisy has similar analysis results.^[3]^ Therefore, sufficiently differentiating tuberculous pleurisy and carefully diagnosing it are necessary. As per our institution review board's policy, ethical approval was not necessary for the case report. Informed consent for publication was given by the patient.

## 2 Case report

A 20-year-old female student had chief complaints of fever and right chest pain. Her past and familial medical history were unremarkable. She had no history of cigarette smoking or alcohol drinking habit, or was on any medication.

The history of the illness was as follows. The patient was originally healthy. In late December 2017, she developed a fever >39°C, dry cough, and right chest pain during inhalation. After 2 days, she visited the outpatient emergency room of our hospital for persistent symptoms.

Blood testing revealed an increased inflammatory reaction. Chest imaging revealed right pleural effusion. The patient was admitted urgently with a diagnosis of right acute bacterial pleurisy. No person she had contact with had similar symptoms or was a patient being treated for tuberculosis.

On admission, her status was as follows: consciousness, clear; height, 152 cm; body weight, 57.6 kg; body mass index, 24.9; body temperature, 38.9°C; blood pressure, 126/83 mmHg; pulse, 107/min and regular; and SpO2, 97% (room air). She had no conjunctival anemia, jaundice, or superficial lymph node swelling. Cardiac sounds were clear. Breathing sounds were reduced in the right lower lung field. She had no abdominal hepatosplenomegaly or edema of either lower limb.

Table 1 presents the findings of the first examination. Blood count results were normal. Biochemistry tests revealed an increased C-reactive protein level (9.1 mg/dL). The serum anti-mycoplasma antibody titer was elevated to 320 times and 128 times on the particle agglutination (PA) test and complement fixation (CF) test, respectively. Urinary pneumococcal and *Legionella* antigen results were negative. The general and acid-fast bacteria results were negative in smears of sucked sputum. The general bacterial culture and human immunodeficiency virus antibody results were negative.

**Table 1:**
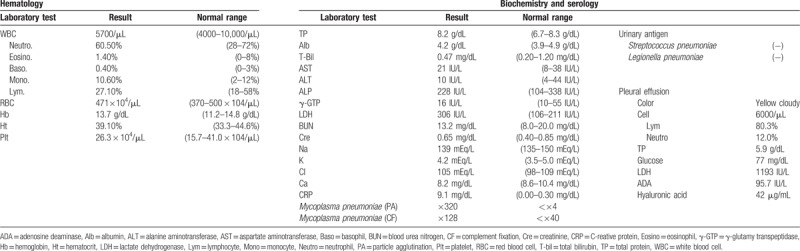
Laboratory findings on admission.

Table 1 presents the results of the pleural effusion, which was yellow and cloudy. The glycoprotein and lactate dehydrogenase levels were 5.9 g/dL and 1193 IU/L, respectively, which indicated an exudative pattern. The white blood cell count was 6000/μL. The cell fractions were 80.3% lymphocytes and 12% neutrophils. Atypical cells were not present. General and acid-fast bacteria smears and the general bacterial culture results were negative.

**Figure 1:**
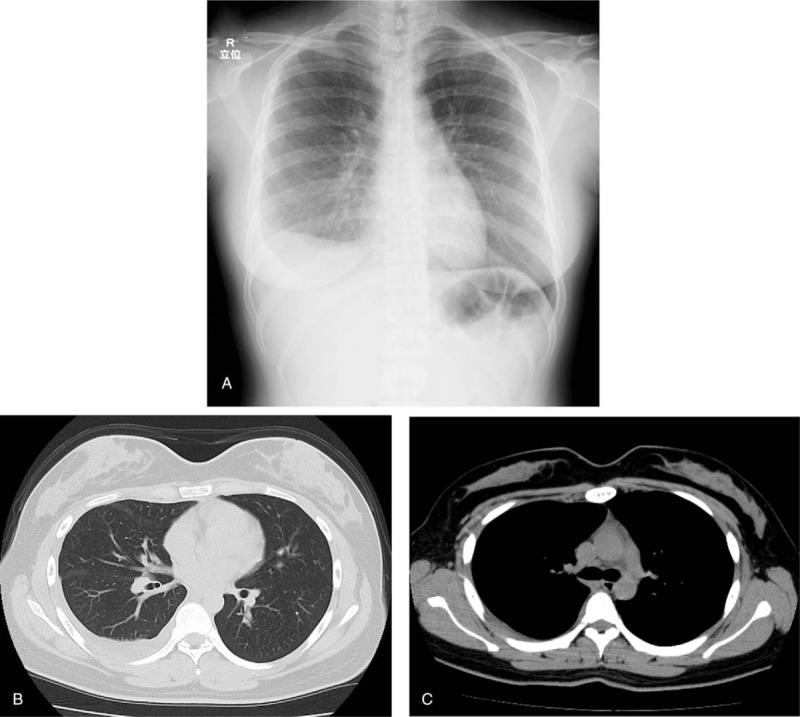
(A) Chest X-ray revealed right pleural effusion. (B), (C) Chest plain computed tomography (CT) revealed right pleural effusion but no intrapulmonary lesion or significant enlargement of the hilar or mediastinal lymph node.

The imaging findings of the first examination are presented in Figure 1. Chest plain radiography revealed right pleural effusion. Chest plain computed tomography (CT) revealed right pleural effusion but no intrapulmonary lesion or significant enlargement of the hilar or mediastinal lymph node.

**Figure 2:**
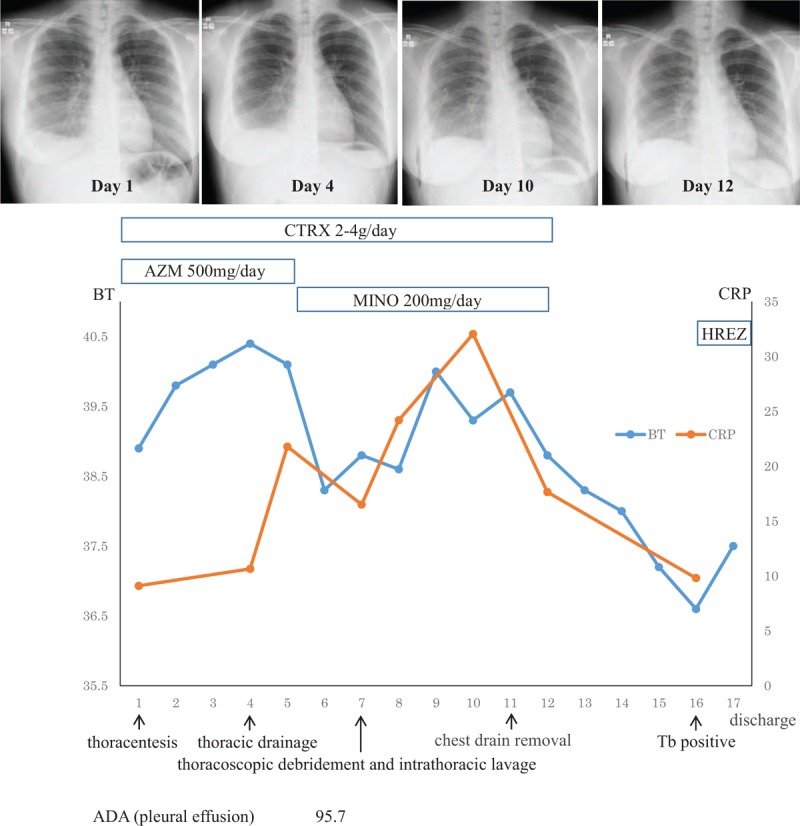
Clinical course of the patient. CTRX: ceftriaxone, AZM: azithromycin, MINO: minocycline, H: isoniazid, R: rifampicin, E: ethambutol, Z: pyrazinamide, Tb: tuberculosis, ADA: adenosine deaminase.

**Figure 3:**
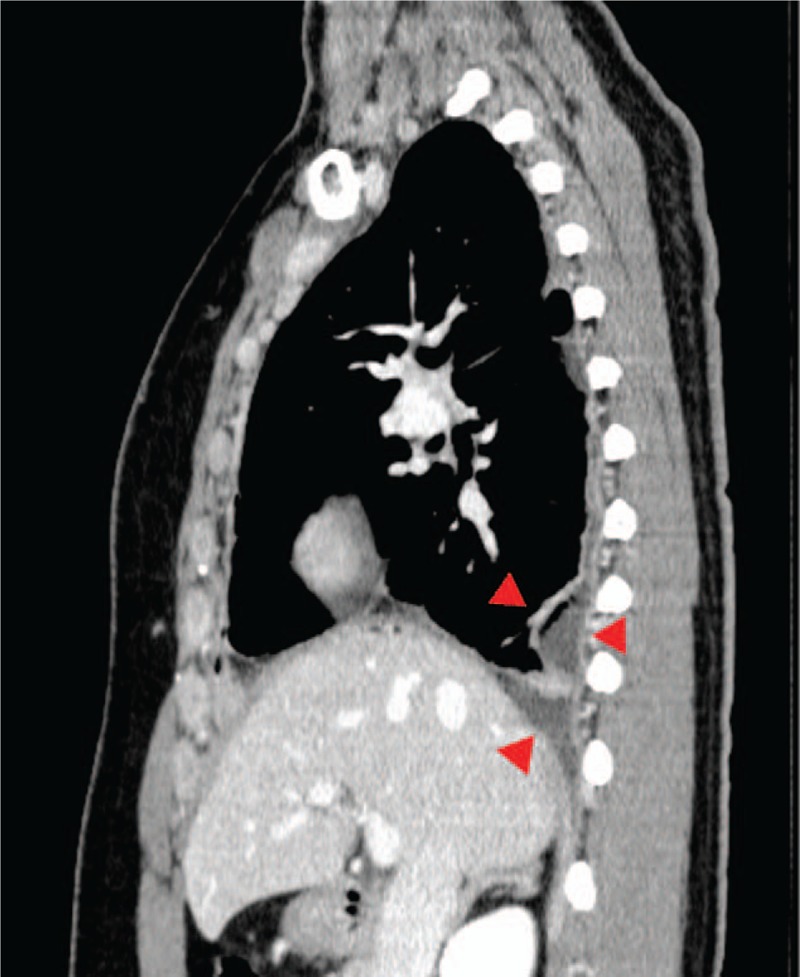
The contrast-enhanced chest CT image shows thickening of the right pleura and residual pleural effusion (arrowheads) on day 6 postadmission.

Figure 2 depicts the patient's course postadmission. After thoracentesis, acute bacterial pleurisy was suspected, based on the patient's clinical course and test findings. Mycoplasma pleurisy was considered, based on the increased anti-mycoplasma antibody titer (PA test, 320-fold; CF test, 128-fold), for which a drip infusion of ceftriaxone and azithromycin was initiated on admission day. The right pleural effusion increased by day 4 postadmission, and was drained. On day 6 postadmission, a high adenosine deaminase (ADA) level (95.7 IU/L) in the pleural effusion was detected. However, this finding is not inconsistent with the features of *M pneumoniae* infection-induced pleural effusion. On day 7 postadmission, surgical thoracoscopic debridement of empyema and thoracoscopic irrigation and drainage were applied to the region with residual pleural effusion (Fig. 3). The lung was covered with a pyogenic pleural membrane and was adhered to the chest wall. After dissecting the adhesion, no specific pleural finding was observed in the thoracic cavity. The residual pleural effusion was not purulent.

The patient's fever declined gradually and the inflammatory reaction decreased. These findings are consistent with the course of *M pneumoniae* infection. However, on day 16 postadmission, *Mycobacterium tuberculosis* was detected in the acid-fast bacterial culture of the pleural effusion, which had been submitted on admission. Based on this finding, acute *M tuberculosis*-induced pleurisy was definitely diagnosed.

**Figure 4:**
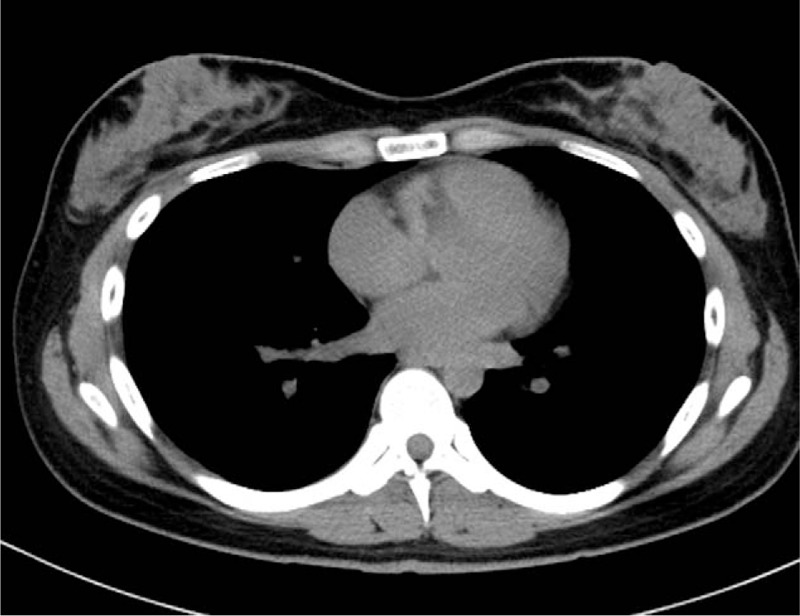
Chest plain CT. The right pleural effusion disappeared and diffuse right pleural thickening improved.

Treatment with 4 antituberculous drugs was initiated on day 16. The drugs were administered for 2 months, followed by the administration of isoniazid and rifampin for 4 months. She completed treatment with no noticeable adverse events, and the right pleural effusion disappeared and diffuse right pleural thickening improved (Fig. 4).

## 3 Discussion

We encountered a patient with tuberculous pleurisy, which acutely developed over 2 days. The onset courses of tuberculous pleurisy and pulmonary tuberculosis differ. Clinicians should be firmly aware of this. Tuberculous pleurisy tends to develop acutely in young people.^[2]^

In the present patient, the clinical course and blood test findings supported a diagnosis of *M pneumoniae* infection. The initial treatment for acute bacterial pleurisy centered on mycoplasma pleurisy. The patient met all 6 items of the criteria proposed by the old Japanese Respiratory Society Guidelines for the Management of Community-acquired Pneumonia for differentiating between atypical pneumonia, represented by *M pneumoniae* pneumonia, and bacterial pneumonia.^[4]^ The sensitivity and specificity for atypical pneumonia are 77.9% and 93%, respectively, when 4 items or more are present. Therefore, we began to suspect more strongly *M pneumoniae* infection. With regard to anti-mycoplasma antibody levels, *M pneumoniae* infection is strongly suspect when the titer in a single serum sample is 320 times or higher and 64 times or higher in the PA and CF tests, respectively.^[5]^ However, for a definite diagnosis, a change in the antibody titer needs to be confirmed using paired sera (eg, an increase of times or greater in the serum antibody titer from the acute phase to recovery phase). However, our judgment was based on only a single serum sample in the present patient; the antibody titer in the recovery phase was not measured.

Investigators have indicated that specific immunoglobulin M (IgM) and immunoglobulin G (IgG) antibodies against *M pneumoniae* are both retained in the circulation for 1 year after a *M pneumoniae* infection.^[6]^ Permanent immunity is not acquired against *M pneumoniae*. Therefore, the bacterium may repeatedly infect humans apparently or latently, and antibodies are produced with each infection. Mycoplasma-specific IgM and IgG antibody carriers accordingly exist at a specific rate in healthy populations.^[7]^ Therefore, an increased level of antibodies in a single serum during the acute phase of *Mycoplasma* infection does not necessarily indicate that the infection is in its acute phase. This factor is a limitation of antibody measurement, although the test is useful. Lymphocyte dominance and a high ADA level in *M pneumoniae* infection-induced pleural effusion, which have been reported,^[3]^ also contributed to the misdiagnosis.

Other than tuberculous pleurisy, representative diseases that may involve lymphocytic pleural effusion are malignant disease, lymphoma, and collagen disease.^[8]^ Cha et al^[3]^ report that *M pneumoniae* should be considered a cause of disease when lymphocyte-predominant pleural effusion exists. In our patient, lymphocytes were the dominant cell fraction and the ADA level was high in the pleural effusion, and anti-mycoplasma antibody results were positive, which served as a red herring. Furthermore, her condition improved without antituberculous drug therapy, although the disease was tuberculous pleurisy.

Some cases of tuberculous pleurisy spontaneously remit.^[9]^ This case may have been included in this group. When the ADA level exceeds 100 IU/L, the disease is likely a malignant disease, empyema, or a lymphoproliferative disease, rather than tuberculous pleurisy.^[10]^ Clinicians should be aware that a high ADA level does not always indicate tuberculous pleurisy.

Only a few cases of *M pneumoniae* infection requiring differentiation between tuberculous pleurisy and mycoplasma pleurisy have been reported in adults.^[11]^ Their courses were not acute, unlike in the present case. The disease developed within only a few days in our patient and differentiation from acute *M pneumoniae* infection was difficult. In a suspected case of acute *M pneumoniae* infection-induced pleural effusion, if lymphocytes are the dominant cell fraction and the ADA level is high in the pleural effusion, it is important to perform an interferon-gamma release assay and polymerase chain reaction test for *M tuberculosis* to investigate the possibility of tuberculous pleurisy while waiting for the acid-fast bacterial culture results.

As the patient was young, the surgeon applied thoracoscopic irrigation and drainage actively to the region with poor drainage of the pleural effusion. This treatment was also aimed at preventing a sequela of bacterial pleurisy: formation of a pyogenic pleural membrane. On reviewing this case, we believe we should have more carefully made this decision for thoracoscopic treatment. In patients with tuberculous pleurisy, only a few cases of iatrogenic pericostal tuberculosis after thoracostomy tube placement^[12]^ and thoracocutaneous fistula formation after several months^[13]^ have been reported. These events suggest that applying unnecessary drainage should be avoided. However, thoracoscopy under local anesthesia is useful for diagnosing tuberculous pleurisy,^[14]^ and is an appropriate treatment when tuberculous pleurisy is actively included in the differential diagnosis.

Another reason for the difficulty in making the correct diagnosis was that the thoracoscopic findings were insufficient. No additional information suggesting tuberculous pleurisy was obtained because pleural changes do not occur early after onset.^[15]^ The patient's internist did not actively request the surgeon to perform a pleural biopsy and acid-fast bacterial test because tuberculous pleurisy was not suspected when she underwent thoracoscopy. This factor should also be considered.

## 4 Conclusion

We report the clinical course of tuberculous pleurisy which was initially diagnosed as acute *M pneumoniae* infection. Clinicians need to be aware of the usefulness and limitations of the anti-mycoplasma antibody test and closely examine the possibility of tuberculous pleurisy before judging that lymphocyte-dominant pleural effusion with a high ADA level is caused by *M pneumoniae* infection.

## Author contributions

**Conceptualization:** Daizo Yaguchi.

**Data curation:** Daizo Yaguchi.

**Formal analysis:** Daizo Yaguchi.

**Investigation:** Daizo Yaguchi, Masao Ito.

**Methodology:** Daizo Yaguchi.

**Project administration:** Masato Shizu, Noriko Inoue, Daisuke Kobayashi, Naoyuki Imai, Masao Ito.

**Resources:** Daizo Yaguchi.

**Visualization:** Daizo Yaguchi.

**Writing – original draft:** Daizo Yaguchi.

**Writing – review & editing:** Daizo Yaguchi, Motoshi Ichikawa.
